# Supplementary material for: MetaQTL: a package of new computational methods for the meta-analysis of QTL mapping experiments
Source: BMC Bioinformatics. 2007 Feb 8;8:49. doi: 10.1186/1471-2105-8-49 (PMC1808479; doi:10.1186/1471-2105-8-49)
Supplement: Additional File 7 — MetaQTL Package : jar file and tutorial. This Zip archive contains both the MetaQTL JAR file and the files of the tutorial. [file 1471-2105-8-49-S7.zip › org.inra.metaqtl/doc/org/metaqtl/adapter/ChromosomeAdapter.html]

ChromosomeAdapter


|  |  |  |
| --- | --- | --- |
| **Overview** \| **Package** \| **Class** \| **Use** \| **Tree** \| **Deprecated** \| **Index** \| **Help** | |  |
| PREV CLASS   **NEXT CLASS** | **FRAMES**    **NO FRAMES**     **All Classes** |  |
| SUMMARY: NESTED \| FIELD \| CONSTR \| METHOD | DETAIL: FIELD \| CONSTR \| METHOD |  |


---


## org.metaqtl.adapter Class ChromosomeAdapter

```
java.lang.Object
  org.metaqtl.adapter.ChromosomeAdapter
```

---

``` public final class ChromosomeAdapter extends java.lang.Object ```

This class defines some usefull methods to adapt `Chromosome`
objects into other kinds of objects or the inverse.

---

**Constructor Summary**

`ChromosomeAdapter()`


| **Method Summary** | |
| --- | --- |
| `static Chromosome` | `toChromosome(IBioLGroup group, IBioCross crossDesign, int mappingFunction, int mappingUnit)`             This method converts a linkage group into a chromosome of type `Chromosome` with the given cross design and the given mapping context. |
| `static Chromosome[]` | `toChromosomes(IBioGenome map)`             This methods converts the given map into an array of `Chromosome` which represents the chromosomes of the input mpe. |
| `static IBioGenome` | `toIBioGenome(Chromosome[] chromosomes, int gout)`             This routine converts an array of chromosomes into a genetic map with as many chromosomes as the length of the array. |

**Methods inherited from class java.lang.Object**

`clone, equals, finalize, getClass, hashCode, notify, notifyAll, toString, wait, wait, wait`

**Constructor Detail**

### ChromosomeAdapter

```
public ChromosomeAdapter()
```


**Method Detail**

### toChromosomes

```
public static Chromosome[] toChromosomes(IBioGenome map)
```

:   This methods converts the given map
    into an array of `Chromosome` which represents
    the chromosomes of the input mpe.

    :   **Parameters:**: `map` - **Returns:**: an array of `Chromosome`

---


### toChromosome

```
public static Chromosome toChromosome(IBioLGroup group,
                                      IBioCross crossDesign,
                                      int mappingFunction,
                                      int mappingUnit)
```

:   This method converts a linkage group into a chromosome of type
    `Chromosome` with the given cross design
    and the given mapping context.

    :   **Parameters:**: `group` - the linkage group to convert.: `crossDesign` - the cross design.: `mappingFunction` - the mapping function.: `mappingUnit` - the mapping unit. **Returns:**: the chromosome

---


### toIBioGenome

```
public static IBioGenome toIBioGenome(Chromosome[] chromosomes,
                                      int gout)
```

:   This routine converts an array of chromosomes into a genetic map
    with as many chromosomes as the length of the array. The second
    argument gout takes its values in [0,2] :

    - 0 - Both marker and QTL are included.
    - 1 - Only marker are included.
    - 2 - Only QTL are included.

    Use the static attributes `MAPPING_UNIT` and
    `MAPPING_FUNCTION` to change the genetic map
    mapping unit and function.

    :   **Parameters:**: `chromosomes` - the array of `Chromosome` to convert.: `gout` - the convertion mode. **Returns:**: a genetic map.


---


|  |  |  |
| --- | --- | --- |
| **Overview** \| **Package** \| **Class** \| **Use** \| **Tree** \| **Deprecated** \| **Index** \| **Help** | |  |
| PREV CLASS   **NEXT CLASS** | **FRAMES**    **NO FRAMES**     **All Classes** |  |
| SUMMARY: NESTED \| FIELD \| CONSTR \| METHOD | DETAIL: FIELD \| CONSTR \| METHOD |  |


---
